# Tension-Band Plating for Infantile Blount Disease: Efficacy and Outcomes Comprehensive Review

**DOI:** 10.7759/cureus.62514

**Published:** 2024-06-17

**Authors:** Daniel Raftis, Jordan Helbing, Sarah Dance, Alana O'Mara, Sean Tabaie

**Affiliations:** 1 Orthopaedic Surgery, The George Washington University School of Medicine and Health Sciences, Washington DC, USA; 2 Orthopaedic Surgery, Children’s National Hospital, Washington DC, USA; 3 Orthopaedic Surgery, Children's National Hospital, Washington DC, USA

**Keywords:** guided growth, tibia vara, epiphysiodesis, tension-band plating, infantile blount’s disease

## Abstract

Infantile Blount disease (IBD) is a pathologic varus knee deformity that, if left untreated, can lead to abnormal gait, limb length discrepancy, and pain. Traditionally, bracing and tibial osteotomy have been the primary treatments. More recently, guided growth with tension-band plating (TBP) has gained popularity, although there is a lack of data stratifying between the infantile, juvenile, and adolescent disease types. Therefore, the present review aims to determine the efficacy and complications of TBP in the IBD population.

A systematic review was conducted following the PRISMA guidelines. Eligible studies included those focused on guided growth correction for IBD. Studies that did not stratify subjects by subgroup (infantile, juvenile, and adolescent) within their analysis were excluded. The outcomes of interest included demographic information, correction rate, failure rate, recurrence rate, and postoperative complications.

Database review identified 541 studies. After screening, seven studies met our inclusion criteria, all of which were retrospective observational studies published between 2012 and 2022. In total, 92 limbs afflicted with Infantile Blount Disease underwent treatment with TBP. The recorded follow-up period ranged from four months to eight years. The age of patients at the time of surgery varied from 1.8 to nine years. On average, there was a 78.99% correction of deformities, with a range of 57.14% to 100%. Six studies provided data on failure and recurrence rates, with an average rate of 23.47%. Notably, infection and hardware failure emerged as the most prevalent postoperative complications, with mean rates of 11.44% and 9.50%, respectively. The average reoperation rate was 29.90%, with a range from 0.00% to 47.06%.

The current literature shows a high rate of deformity correction with a relatively low risk of complications after TBP for IBD. Given the reported reoperation rates greatly varied, further data is needed to determine risk factors for reoperation following TBP. Our results suggest that guided growth with TBP may be a preferable first-line treatment for IBD.

## Introduction and background

Infantile Blount Disease (IBD), also known as pathologic tibia vara, is a progressive pathologic genu varum deformity. While the etiology is poorly understood, a defect in the medial proximal tibial physis leads to the development of progressive genu varum, procurvatum, and internal rotation of the tibia [1-3]. Disease progression is associated with the commencement of walking and increasing body mass, with the incidence of IBD in the United States increasing with rising rates of childhood obesity [4]. IBD is diagnosed before the age of four years and typically affects both legs. In addition to infantile type, Blount disease may also be classified as juvenile or adolescent. The onset of adolescent Blount disease typically occurs after 10 years of age and is typically unilateral compared to bilateral as in IBD. Juvenile Blount disease is usually classified as neglected cases of IBD found in children aged four to 10 years old [5]. While all forms of Blount disease result in progressive genu or tibia varum, the infantile type is the most severe form and different treatment strategies may be employed in younger children than their adolescent counterparts. Table 1 outlines the discrepancies between all types of Blount Disease.

**Table 1:** Characteristics Differentiating Between Blount Disease Types

	Infantile Blount	Juvenile Blount	Adolescent Blount
Etiology	Unknown but linked to mechanical overload and obesity	Unknown but linked to undiagnosed infantile-type and obesity	Unknown but linked to obesity
Age at Diagnosis	Before 4 years	Between 4-10 years	After 10 years
Laterality	50% Bilateral	Bilateral	Unilateral
Classification	Langenskiold	None	None
Severity	Most severe form	Intermediate severity	Least severe form
Physical Exam	Genu varus, flexion, internal rotation, compensatory distal femoral valgus	Progressive genu varus, more severe physeal irregularity compared to the adolescent-type	Progressive genu varus, distal femoral varus, distal tibia valgus
Treatment	Bracing +/- Surgery	Surgery only	Surgery only

Traditional management of IBD involves conservative management with knee-ankle-foot orthoses (KAFO) braces before three years of age. Braces can present as challenging for patients and parents as they need to be worn during all waking hours, locked in extension when the patient is standing, and unlocked to allow flexion when seated. Additionally, bracing can be cumbersome for more obese patients, which the majority of IBD patients are [5]. However, it is important to note that there are no clear guidelines based on the current literature on the daily wear of the KAFOs as well as how long the braces should be worn [6-9]. Furthermore, there is no consensus regarding the efficacy of bracing as it generally is reserved for IBD patients with mild disease that may or may not spontaneously resolve over time [10,11]. The successful correction with bracing has been correlated to the degree of angulation at the commencement of treatment [9]. If patients fail treatment with bracing, then surgical management involving proximal tibial osteotomies is generally recommended. Unfortunately, proximal tibial osteotomy, while effective, is invasive and carries a significant recovery with risks of deformity recurrence, compartment syndrome, and infection [12]. In cases of severe deformity and ample growth remaining, physeal bar resection may be indicated with or without osteotomy.

In a review of the literature, minimally invasive guided-growth management with tension-band plating (TBP) has gained popularity in managing angular knee deformities; however, unlike in juvenile or adolescent Blount’s disease, a consensus has yet to be made regarding its use and efficacy in IBD. Available literature tends to have small sample sizes or combined analysis of the complications and efficacy of TBP in infantile, juvenile, and adolescent Blount’s Disease making conclusions difficult to draw. The objective of the present study is to determine the efficacy and complications of TBP in the treatment of IBD through a systematic review of the literature, evaluating for rate of correction, failure rate, reoperation rate, and postoperative complications.

## Review

Materials and methods

The systematic review pulled studies from PubMed, Scopus, and Cochrane Central, focusing on guided growth in Infantile Blount’s disease. Institutional Review Board (IRB) approval was not required for the study. The official search within these databases was carried out in September of 2023. See Appendix 1 for the entire search strategy. This review only included randomized controlled trials or prospective/retrospective observational studies that were written in English, focused on either Blount's Disease or Tibia Vara, and included the infantile subgroup (1-3 years) within its analysis. Studies were included if they reported outcomes after guided growth or its synonyms which included: hemiepiphysiodesis, tension-band plating, and eight plates. All studies that used animal subjects, systematic reviews, meta-analyses, or technique papers were excluded. Papers that did not provide subgroup analysis for IBD patients or did not use guided growth or its synonym for surgical correction were excluded.

Covidence (Melbourne, Australia), a third-party resource, was used for data extraction. After the removal of duplicated studies, two independent reviewers (DR and JH) screened articles using the above-mentioned criteria and extracted data into a spreadsheet for further analysis. The methodological quality of each study was also assessed by each reviewer to reduce the risk of bias using the Newcastle-Ottawa Scale or Risk of Bias Assessment Tool for Nonrandomized Studies. Only studies that reached a consensus among reviewers were included. All studies that did not reach an initial consensus were reviewed for a second time together, and each inclusion and exclusion criteria was explicitly identified. If consensus was not met following the second review, the senior author (DR) settled the disagreement.

The outcomes of this review included duration of follow-up, rate of correction, rate of failure/recurrence, rate of reoperation, and postoperative complications, such as infection, hardware failure, and overcorrection. For this study, the duration of follow-up was reported in either months or years and all other results were reported as either whole numbers or percentage of total population. The preoperative and postoperative measurements used were also recorded for each study and included in our analysis.

Results

**Figure 1:**
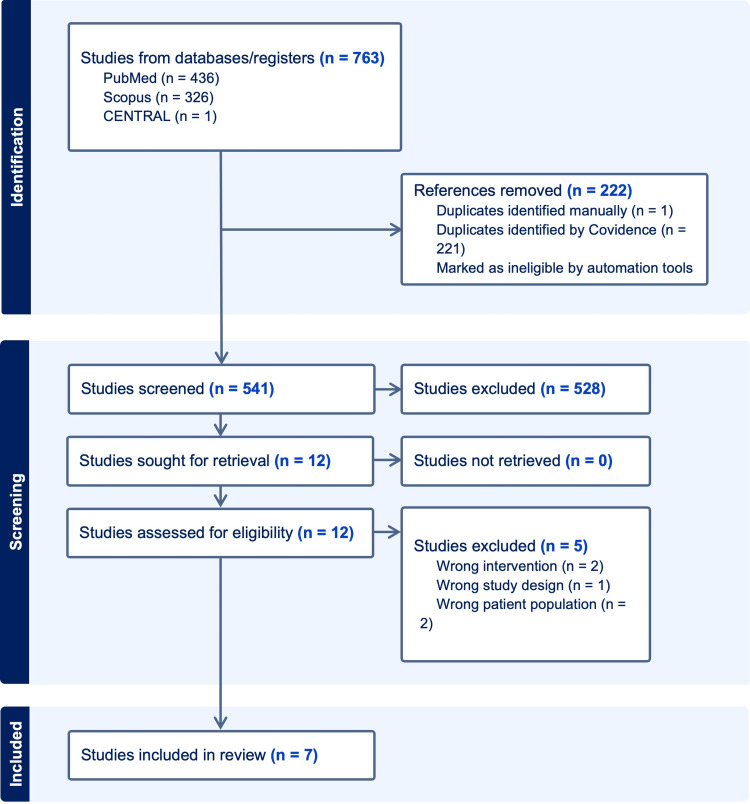
Preferred Reporting Items for Systematic Reviews and Meta-Analyses (PRISMA) Flow Chart Showing Inclusion and Exclusion Criteria

The initial database search resulted in 763 abstracts. After removing 222 duplicates and applying our inclusion and exclusion criteria, our abstract screening concluded with 12 potentially relevant papers that underwent full article review for eligibility. Of these 12 studies, five were excluded for either incorrect intervention, wrong patient population, or wrong study design. Thus, seven studies were included in our analysis (Figure 1).

Study Characteristics and Patient Demographics

All seven studies included in the study were retrospective and published between 2012 and 2022. Three studies were conducted in the United States (US), and one from each respective country: Egypt, Israel, Benin, and South Africa. A total of 92 limbs underwent surgical correction. Of the five studies that included sex in their demographics, there was found to be an equal male-to-female ratio. Obesity was also tracked by five of the seven studies but was measured using three different methods. Scott et al. and Griswold et al. reported age-based body mass index (BMI) percentile with an average of 93.76% between the two studies [12,13]. Together, the average BMI of included patients in Assan et al. and Heflin et al. was 20.8 [1,14]. Finally, Maré et al. reported that two of the seven patients in their study had obesity [15]. The age at the time of surgery was included in six of the studies, which combined had a mean age of 4.14 years, ranging from 1.8 to 8.7 years. Five studies reported a length of follow-up, with an average duration of 3.2 years (Table 2).

**Table 2:** Demographics and Study Details for Each Included Study SD: standard deviation; BMI: body mass index; - data not reported.

First Author	Affected Limbs, N	Publication Year	Country	Study Design	Age at Surgery in Years, Mean (Range)	Follow-Up, Mean (Range)	Sex Ratio, Male:Female	BMI or Rate of Obesity, Mean + SD (Range)
Khanfour [3]	17	2021	Egypt	Case series	4.2 (3-7)	4.8 (2-8) years	5:12	N/A
Scott [12]	18	2012	United States	Case series	4.8 (2.8-8.7)	N/A	N/A	94.42% (75-97)
Danino et al. [2]	11	2020	Israel	Cohort	Before age 4	N/A	N/A	N/A
Griswold et al. [13]	17	2020	United States	Cohort	3.25 (+/- 1.4)	4.31 years	6:11	93.1% (+/-14.5) for age
Assan et al. [1]	14	2021	Benin	Cohort	6.5 (3-9)	24 (15-27) months	4:6	25.3 +/- 3.1
Heflin et al. [14]	8	2016	United States	Cohort	2 (1.8-2.4)	26.67 (14-43) months	4:2	16.25 (11.9-19.9)
Maré et al. [15]	7	2022	South Africa	Case series	4.1 (2.9-5.8)	32 (17-62) months	4:3	2 with obesity
Total	92	-	-	-	-	-	23:23	-
Mean Rate	-	-	-	-	4.14	3.2 years	-	-

Measurements Used for Severity of Deformity and Degree of Correction

Amongst the seven studies included, 11 unique preoperative and postoperative measurements were used to quantify the severity of deformity and degree of correction (Table 3). Meta-diaphyseal angle (Drennan angle) and mechanical axis deviation (MAD) were included in four of the studies. Tibiofemoral angle (HKA), mechanical medial proximal tibial angle (MMPTA), and mechanical lateral distal femoral angle (mLDFA) were measurements used in two studies. Five other unique measurements were only found to be in individual studies. Of the four studies that included the Drennan angle, two used it for preoperative measurement without postoperative comparison. Similarly, the medial physeal slope (MPS) was only included as a preoperative measurement in one study. All remaining measurements were used both preoperatively and postoperatively within the respective studies.

**Table 3:** Inclusion of Different Radiographic Measurements for Both Preoperative and Postoperative Analysis by Each Author HKA: tibial femoral angle; MMPTA: mechanical medial proximal tibial angle; mLDFA: mechanical lateral distal femoral angle; Drennan Angle:  metadiaphyseal angle, MAD: mechanical axis deviation; m-FTVA: mechanical-femoral tibial varus deformity; ITT: internal tibial torsion; MPD: medial plateau depression; MPS: medial physeal slope; * only measured preoperatively; - data not reported.

Measurement	Khanfour [3]	Scott [12]	Danino et al. [2]	Griswold et al. [13]	Assan et al. [1]	Heflin et al. [14]	Maré et al. [15]	Total
HKA (TFA)	-	-	-	-	X	-	X	2
MMPTA	-	-	X	-	X	-	-	2
mLDFA	-	-	X	X	-	-	-	2
Drennan Angle (MDA)	-	X*	-	X	-	X	X*	4
MAD	-	X	-	X	-	X	X	4
m-FTVA	X	-	-	-	-	-	-	1
ITT	X	-	-	-	-	-	-	1
MPD	X	-	-	-	-	-	-	1
distal femoral deformity	X	-	-	-	-	-	-	1
MPS	-	-	-	-	-	-	X*	1

Rate of Correction

The rate of correction was calculated in all seven studies and was found to be an average of 78.99%. The rate of failure/recurrence and reoperation was calculated from six studies. The average failure/recurrence rate was 23.47% and the reoperation rate was 23.47%. Of note, Griswold et al. reported 24 reoperations for eight extremities that demonstrated deformity recurrence [13]. Overcorrection was measured in four studies and found to occur at an average rate of 5.04%. The rate of correction was the only outcome collected from Danino et al. due to a lack of subgroup analysis [2]. Additional outcomes regarding the rate of correction can be found in Table 4.

**Table 4:** Post-Surgical Outcomes and Complications following Tension-Band Plating in Infantile Blount Disease - data not reported.

First Author	Affected Limbs, N	Correction, N (Rate)	Failure/ Recurrence, N (Rate)	Reoperation, N (Rate)	Post-op Infection, N (Rate)	Hardware Failure, N (Rate)	Overcorrection, N (Rate)	Other Complications, N (Rate)
Khanfour [3]	17	16 (94.11%)	1 (5.88%)	1 (5.88%)	0 (0.00%)	1 (5.88%)	-	-
Scott [12]	18	16 (88.89%)	2 (11.11%)	1 (5.56%)	3 (16.67%)	3 (16.66%)	0 (0.00%)	-
Danino et al. [2]	11	7 (63.64%)	-	-	-	-	-	-
Griswold et al. [13]	17	12 (70.59%)	8 (47.06%)	8 (47.06%)	5 (29.1%)	1 (5.88%)	1 (5.88%)	6 (35.29%)
Assan et al. [1]	14	11 (78.57%)	3 (21.43%)	2 (14.29%)	-	2 (14.29%)	-	-
Heflin et al. [14]	8	8 (100%)	1 (12.5%)	1 (12.5%)	0 (0.00%)	0 (0.00%)	0 (0.00%)	-
Maré et al. [15]	7	4 (57.14%)	3 (42.86%)	0 (0.00%)	-	1 (14.29%)	1 (14.29%)	1 (14.29%)
Mean Rate	-	78.99%	23.47%	29.90%	11.44%	9.50%	5.04%	24.79%

Postoperative Complications

Postoperative complications were also collected from each study (Table 4). Postoperative infection, hardware failure, and overcorrection were the three most included complications. For the four studies that measured postoperative infection, the average rate of infection was found to be 11.44%. Of the six studies that included hardware failure, the average rate was calculated to be 9.50%. Both Griswold et al. and Mare et al., measured additional complications which were collectively averaged in the “other” category to be 24.79% [13,15].

Discussion

This systematic review supports the notion that TBP demonstrates a promising correction rate in IBD. The observed average correction rate of 78.99% across all studies signifies a substantial improvement in the deformity. Such efficacy is crucial, especially considering the challenges posed by traditional approaches. Bracing has been recommended as an option for the non-surgical management of IBD, but its efficacy has yet to be established by a controlled trial [10,16].

Tibial osteotomy has historically been the established treatment strategy for surgical correction, but it is associated with high risks of recurrence varying from 40-70% [17-20]. In comparison, the average rate of failure or recurrence within the six TBP studies analyzed was found to be 23.47% (Range 5.88-47.06%). Recurrent deformity results in the need for additional procedures placing patients at greater risk for complications [19]. Alternatives to tibial osteotomy like TBP have become more common recently as pediatric orthopedic surgeons search for definitive treatment for their patients with IBD.

Despite the promising correction rates of TBP, it is imperative to note the associated complications of each study. The identified average rates of postoperative infection (11.44%), hardware failure (9.50%), reoperation (29.90%), and recurrence (23.47%) highlight the need for caution. Notably, the reoperation rate's wide range from 0% to 47.06% signifies varying experiences among different studies. Griswold et al. reported the highest reoperation rate of the studies at 47.06% [13]. It is also important to note their reoperation rate calculation was based on the total number of reoperation procedures, not the number of limbs that required reoperation, which is what this study utilized for calculating reoperation rate. Furthermore, in their discussion, they note that 100% of the reoperations were found within children who were Langenskiold stage 3 or greater. In the management of Blount's disease by osteotomy, the Langenskiold stage at the time of treatment is a critical determinant of therapeutic success [19]. Griswold et al., concluded that patients with Langenskiold stage less than 2 exhibit a markedly higher rate of treatment success after TBP [13]. This contrasted starkly with outcomes in patients presenting with a Langenskiold stage greater than 3, who not only had a high recurrence of deformity but also necessitated additional surgeries such as physeal bar resection. Supporting this, Heflin et al. further elucidate that children at lower Langenskiold stages (specifically below stage 5) achieve more reliable correction with a significantly reduced rate of recurrence [14]. These insights collectively underscore the necessity of early intervention in Blount's disease, where lower Langenskiold stages are associated with more favorable outcomes and less invasive subsequent interventions.

In the surgical management of IBD with TBP, surgical technique, and hardware failure have emerged as prevalent and impactful complications. Screw breakage, amongst other forms of hardware failure, can lead to treatment failure and increase the need for reoperation. In their analysis, Scott et al., found that all three instances of screw breakage involved cannulated titanium screws [12]. This finding signals potential weaknesses in the material and design of these screws, prompting Scott et al. to suggest alternatives, such as solid screws, a double screw, or plate configuration, to reduce the risk of breakage. Furthermore, Mare et al. highlight cases where surgeon error led to a loss of fixation, emphasizing the critical role of surgical precision, while Assan et al. point out a higher frequency of screw pull-out or breakage in obese patients, indicating the influence of patient-specific factors [1,15]. Collectively, these findings underscore the need for careful reconsideration of both hardware selection and surgical techniques to optimize outcomes and minimize complications in the treatment of Blount’s disease.

The limited number of studies and their retrospective nature pose inherent limitations. The majority of the included studies were retrospective case series and cohort studies, and all had relatively small sample sizes (Table 2). Conducting prospective studies with larger cohorts specifically focusing on IBD would strengthen the evidence base and provide more definitive conclusions regarding TBP's efficacy and safety in this particular subgroup. Each of the included studies also used its unique combination of radiographic measurements as they preoperatively define deformity and postoperatively determine the rate of correction (Table 4). Due to the lack of measurement consistency, it is difficult to accurately compare the correction, reoperation, and complication rates between studies. Creating a consensus for preoperative and postoperative measurements for the surgical management of IBD would improve this field of literature and is a necessary step going forward for future large-scale studies.

Despite these limitations, the collective evidence suggests that TBP holds promise as a primary treatment for IBD. Its relatively high correction rates and manageable complication profile advocate for further exploration and refinement of surgical techniques and patient selection criteria. Additionally, comparative studies directly contrasting TBP with traditional treatments like bracing and osteotomy could offer deeper insights into its superiority or complementarity in managing IBD.

## Conclusions

The current systematic review significantly contributes to understanding TBP's role in managing IBD, recognizing its promising outcomes while acknowledging the associated complexities and research gaps. TBP was found to have relatively high correction and low complication rates, which advocates for its use in IBD. It also aids clinicians in informed decision-making. The relatively low number of studies included in the present review highlights the ongoing need for further investigation and refinement of the surgical approach in treating IBD with TBP.

## Appendices

Appendix 1 

**Table 5:** Search Strategy for Covidence

Database	Search Strategy
PubMed	(infan* [tiab] OR infant [mesh] OR baby [tiab] OR babies [tiab] OR newborn* [tiab] OR new born* [tiab] OR toddler* [tiab] OR child, preschool [mesh]) AND (blount* [tiab] OR tibia vara [tiab] OR bow* leg* [tiab] OR bowleg* [tiab] OR bow-leg* [tiab] OR bowed-leg* [tiab] OR genu var* [tiab] OR genu varum [mesh] OR varus deformit* [tiab] OR osteochondrosis [tiab] OR osteochondrosis [mesh] OR deformans tibia* [tiab]) AND (guided growth [tiab] OR hemiepiphys* [tiab] OR epiphys* [tiab] OR tension band* [tiab] OR plate* [tiab] OR eight-plate* [tiab] OR TBP [tiab] OR osteotom* [tiab] OR osteotomy [mesh] OR staple* [tiab] OR suture* [tiab] OR sutures [mesh] OR phemister [tiab])
Scopus	(TITLE-ABS-KEY (infan*) OR TITLE-ABS-KEY (baby) OR TITLE-ABS-KEY (babies) OR TITLE-ABS-KEY (newborn*) OR TITLE-ABS-KEY (“new born*”) OR TITLE-ABS-KEY (toddler*)) AND (TITLE-ABS-KEY (blount*) OR TITLE-ABS-KEY (“tibia vara”) OR TITLE-ABS-KEY (“bow* leg*”) OR TITLE-ABS-KEY (bowleg*) OR TITLE-ABS-KEY (“bow-leg*”) OR TITLE-ABS-KEY (“bowed-leg*”) OR TITLE-ABS-KEY (“genu var*”) OR TITLE-ABS-KEY (“varus deformit*”) OR TITLE-ABS-KEY (osteochondrosis) OR TITLE-ABS-KEY (“deformans tibia*”)) AND (TITLE-ABS-KEY (“guided growth”) OR TITLE-ABS-KEY (hemiepiphys*) OR TITLE-ABS-KEY (epiphys*) OR TITLE-ABS-KEY (“tension band*”) OR TITLE-ABS-KEY (plate*) OR TITLE-ABS-KEY (eight-plate*) OR TITLE-ABS-KEY (TBP) OR TITLE-ABS-KEY (osteotom*) OR TITLE-ABS-KEY (staple*) OR TITLE-ABS-KEY (suture*) OR TITLE-ABS-KEY (phemister))
Cochrane (CENTRAL)	((“infan*”) OR (“baby”) OR (“babies”) OR (“newborn*”) OR (“new born*”) OR (“toddler*”)) AND ((“blount*”) OR (“tibia vara”) OR (“bow* leg*”) OR (“bowleg*”) OR (“bow-leg*”) OR (“bowed-leg*”) OR (“genu var*”) OR (“varus deformit*”) OR (“osteochondrosis”) OR (“deformans tibia*”)) AND ((“guided growth”) OR (“hemiepiphys*”) OR (“epiphys*”) OR (“tension band*”) OR (“plate*”) OR (“eight-plate*”) OR (“TBP”) OR (“osteotom*”) OR (“staple*”) OR (“suture*”) OR (“phemister”)) ((infan*) OR (baby) OR (babies) OR (newborn*) OR (“new born*”) OR (toddler*)) AND ((blount*) OR (“tibia vara”) OR (“bow* leg*”) OR (bowleg*) OR (“bow-leg*”) OR (“bowed-leg*”) OR (“genu var*”) OR (“varus deformit*”) OR (osteochondrosis) OR (“deformans tibia*”)) AND ((“guided growth”) OR (hemiepiphys*) OR (epiphys*) OR (“tension band*”) OR (plate*) OR (“eight-plate*”) OR (TBP) OR (osteotom*) OR (staple*) OR (suture*) OR (phemister)).
